# Continuously high *Wolbachia* incidence in flea populations may result from dual-strain infections with divergent effects

**DOI:** 10.1038/s41598-025-09403-2

**Published:** 2025-07-01

**Authors:** Ron Flatau, Aleksandra I. Krawczyk, Michal Segoli, Jeffrey E. Barrick, Hadas Hawlena

**Affiliations:** 1 Jacob Blaustein Center for Scientific Cooperation, The Jacob Blaustein Institutes for Desert Research, Ben-Gurion University of the Negev, Midreshet Ben‑Gurion, Israel; 2 Mitrani Department of Desert Ecology, The Swiss Institute for Dryland Environmental and Energy Research, The Jacob Blaustein Institutes for Desert Research, Ben-Gurion University of the Negev, Midreshet Ben-Gurion, Israel; 3 Department of Molecular Biosciences, The University of Texas at Austin, Austin, TX USA; 4 Present Address: Department of Infection and Immunity, Luxembourg Institute of Health, Esch-sur-Alzette, Grand Duchy of Luxembourg

**Keywords:** Differential effects, Fitness advantage, Fleas, Male-killing, Multiple infections, Reproductive manipulation, Strain coinfection, Superinfection, Vertical cotransmission, *Wolbachia*, Community ecology, Microbial ecology

## Abstract

The continuously high incidence of some endosymbionts in arthropods despite potential conflicts with their hosts is often explained by obligatory relationships, in which the host is fully dependent on its endosymbiont, fitness advantages conferred on hosts by facultative endosymbionts, or reproductive manipulation of hosts by endosymbionts (typically facultative). Yet continuously endosymbiont high incidence is sometimes observed without clear evidence supporting any of these mechanisms. This situation could potentially be explained by the presence of several coinfecting strains of the same endosymbiont species, each affecting the host differently such that their effects counteract one another when studied collectively. Here, we investigated *Wolbachia* endosymbionts of fleas, which stably persist in high loads in all females, with no indication that any of the above mechanisms explain their continuously high incidence. We sequenced fleas and identified two *Wolbachia* strains, designated as *w*Sc1 and *w*Sc2. We then correlated the strain composition in fleas with measures of their reproductive success. We found that fleas with high *w*Sc1 and low *w*Sc2 loads had a higher reproductive success than fleas that had high loads of both strains, low loads of both strains, or no *Wolbachia*, suggesting that *w*Sc1 may provide a direct fitness advantage to their hosts. Conversely, the number of males and total offspring was negatively correlated with *w*Sc2 levels, supporting male killing. Our research demonstrates that endosymbionts’ continuously high incidence may persist through intricate relationships in nature.

**Supplementary Information:**

The online version contains supplementary material available at 10.1038/s41598-025-09403-2.

## Introduction

Endosymbiont incidence in arthropod host populations depends on factors such as endosymbiont transmission modes, interactions with the hosts and other endosymbionts, and effects on the host behavior and reproduction, all of which are environmentally influenced^1^. However, in many cases, despite potential conflicts with their hosts and fluctuating ecological conditions, there exists a continuously high incidence of endosymbionts in arthropod host populations^2–4^. Consistently high incidence is mostly explained by three types of mechanisms^5,6^. The first involves obligatory relationships, in which the host’s survival, reproduction, or both are entirely dependent on its endosymbiont^1,7^. The second mechanism can occur even when the host is not fully reliant on the endosymbiont and involves direct fitness benefits. These advantages may include nutrient supplementation or protection from predators or harsh environmental conditions, provided by facultative endosymbionts under certain conditions^8–12^. The third type of mechanism is reproductive manipulation by the endosymbiont (typically facultative), which enhances its transmission^13^. Reproductive manipulation may distort the sex ratio of arthropod hosts, for example, when they provide an advantage to female offspring over their non-transmitting male siblings by inducing male killing, parthenogenesis, or feminization. The former phenomenon leads to males’ death, reducing the total offspring number^14^, and the latter turns genetic males into fertile phenotypic females with functional ovaries^15^. Another type of reproductive manipulation that does not distort the host sex ratio is cytoplasmic incompatibility (CI), where the offspring of the cross between infected males and uninfected females fail to develop, thereby providing a reproductive advantage to infected females^16^.

Nevertheless, there are cases of continuously high endosymbiont incidence with unclear drivers (e.g^17–20^). One such unusual case is *Wolbachia* bacteria, which stably persist in high loads in all female *Synosternus cleopatrae* fleas but not in males, where they occur only sporadically in low loads^21^. Moreover, despite the endosymbiont being found in all female fleas, we could not find any experimental indications of obligatory relationship, facultative fitness advantage, or reproductive manipulation associated with the endosymbiont^6^. An alternative explanation for the continuously high incidence of *Wolbachia* and other endosymbionts in their arthropod hosts with unclear drivers could be that there is coinfection by multiple endosymbiont strains of the same species, a situation that exists in other flea systems^22^. These strains may have distinct and sometimes opposing effects on their host^23^, which may become obscured when they are studied collectively. Moreover, it is possible that one of the strains damages male hosts, leading to the sex-bias endosymbiont incidence that is observed in this system.

Two lines of evidence suggest that coinfection can solve at least some of the unexplained cases of continuously high endosymbiont incidence. First, the coinfection of arthropods by multiple endosymbiont strains is common in nature (e.g^23–27^). Second, recent genomic analyses^22,28,29^, supported by a handful of experiments^30–35^, provide some evidence for the distinct effects of coinfecting endosymbiont strains on their shared host. These include intriguing experimental examples of *Wolbachia* strain-specific effects in mosquitoes, moths, flower bugs, wasps, *Drosophila*, and butterflies^30–34,36–39^. Thus, we aimed to determine whether differential effects of coinfecting *Wolbachia* strains could explain their continuously high *Wolbachia* incidence in female fleas, despite the lack of experimental support for any of the three proposed mechanisms.

Here we combined field sampling, various molecular techniques, and new analyses of data from a previous experiment that manipulated *Wolbachia* infection to address the following three, non-mutually exclusive, hypotheses: (H1) *S. cleopatrae* fleas are coinfected by multiple *Wolbachia* strains, (H2) these include strains that confer direct fitness advantages, or (H3) cause sex-distortion reproduction manipulations in fleas. We found evidence of coinfections of two *Wolbachia* strains in both male and female *S. cleopatrae*, with initial indications that one of these strains confers a direct fitness advantage to the host, while the other induces male killing. These results suggest that contrasting effects of two coinfecting endosymbiont strains may explain how they collectively have a continuously high incidence in host populations.

## Materials and methods

### Study approach and rationale

The study included multiple steps described in Supplementary Materials, Fig. S1. To test H1, which postulates that the *S. cleopatrae* fleas are coinfected by multiple *Wolbachia* strains, we explored *Wolbachia* genetic variation in *S. cleopatrae *fleas collected from the field and a laboratory colony (Supplementary Text S1;^21^). To do this, we subjected the flea DNA extracts to next-generation multilocus sequence typing (NGMLST), targeting *Wolbachia*’s five housekeeping genes. In most of the fleas, we found two variants per gene. We further assessed whether in these coinfected fleas, there are only two or more—due to possible recombination—*Wolbachia* variants. We used three strategies to address this question. First, we conducted whole-genome sequencing (WGS) of two flea pools, each including a unique combination of variants across genes but only one variant per gene. Second, on the same flea extracts, we conducted three quantitative real-time PCR (qPCR) assays that targeted the *fbpA* gene, using either general primers that are designed to amplify all *Wolbachia* bacteria (hereafter *Wolbachia-*general) or primers that are specific for each variant. We then compared the *Wolbachia*-general load and the summed load of the two variant-specific assays. Third, we compared the structures of five phylogenetic trees, each constructed based on the sequences of a single MLST gene. In the final step, we created a phylogenetic tree of concatenated MLST sequences, which allowed us to explore evolutionary relationships between the two *Wolbachia* variants in *S. cleopatrae* and in relation to six documented *Wolbachia* supergroups. Altogether, the results of the above steps suggest that two *Wolbachia* strains, designated as *w*Sc1 and *w*Sc2, coinfect *S. cleopatrae* fleas (see Results).

To test H2 and H3 regarding the distinct effects of *w*Sc1 and *w*Sc2 on the fleas, we subjected the flea DNA extracts, which were collected during our previous *Wolbachia *infection manipulation (Supplementary Text S2^6^;), to strain-specific qPCRs. In short, in the previous study, measures of reproductive success were quantified for four groups of *S. cleopatrae*. These fleas all originated from the same colony stock but had different combinations of *Wolbachia* status (*Wolbachia*-free obtained by antibiotic treatment or *Wolbachia-*positive) and physiological age (females fed on rodents for either five or ten days^6^;). More details about the study design of this experiment are provided in the Supplementary Text S2. To test H2 regarding the distinct fitness advantages of the two strains, we correlated their quantities with the fleas’ integrated reproductive success index. Similarly, to test H3 regarding distinct reproductive manipulations of the two strains, we correlated the strain loads with the number of female and male flea offspring (Statistical Analyses). Once we found evidence for reproductive manipulation, we tested whether the mechanism was more likely to be male killing or feminization by correlating the quantities of the two strains and the total number of offspring. A decrease in male offspring with no change in the total number of offspring would indicate feminization^40^. In contrast, a simultaneous reduction in the number of male offspring and the total number of offspring would indicate male killing^41^. Note that we used both *Wolbachia*-free and *Wolbachia*-positive groups for some of the analyses testing H2, but only the latter group for testing H3.

### Sources of field-collected and laboratory fleas

*S*. *cleopatrae* fleas were collected during a field survey in 2011 at three sites across the northwestern Negev Desert’s sands in Israel^42^. In parallel, *S. cleopatrae* fleas were bred under laboratory conditions on *G. andersoni* and *G. pyramidum* rodents^6^. More details are provided in Supplementary Text S1.

### DNA extraction

Fleas used in this study were preserved in 70% ethanol before DNA extraction. DNA was extracted from individual fleas using the QIAGEN DNeasy Blood and Tissue Kit. A negative control extraction was included in each extraction session and all the following analyses.

### Next-generation multilocus sequence typing (NGMLST)

We conducted NGMLST analysis on DNA extracts of 128 female fleas: 96 were collected from field-trapped rodents and 32 from the laboratory colony (Supplementary Text S1). We targeted five housekeeping genes commonly used in *Wolbachia* phylogenetic analysis (Table 1;^43^). These genes were amplified from each flea sample using PCR and were evenly pooled based on Qubit fluorometer measurements. The resulting 128 pooled samples were subjected to next-generation sequencing. Paired-end 2 × 300 bp reads were generated on the Illumina MiSeq platform at the DNA services facility of the Research Resources Center at the University of Illinois Chicago.

**Table 1:** *Wolbachia* primers for all molecular assays. The assays include next-generation multilocus sequence typing (NGMLST), polymerase chain reaction (PCR), and real-time quantitative PCR (qPCR). The primers are organized according to the analysis aim and the target gene. Exact sequences and amplicon sizes are indicated. For the NGMLST, the binding regions are indicated in bold. The reaction conditions are provided in the supplementary text S3.

Aim	Gene	Forward primer	Reverse primer	Size (bp)
NGMLST	*gatB*	5’-**ACACTGACGACATGGTTCTACA** GAKTTAAAYCGYGCAGGBGTT-3’	5’-**TACGGTAGCAGAGACTTG** **GTCT**TGGYAAYTCRGGYAAA GA-3’	447
	*coxA*	5’-**ACACTGACGACATGGTTCTACA** AGTCACTATATTCAATATGCGTGCAAAAGGT-3’	5’-**TACGGTAGCAGAGACTT** **GGTCT**RCCAGTTATAACGCC AATAAAAATTGTTGTAG-3’	454
	*hcpA*	5’-**ACACTGACGACATGGTTCTACA** GGCTGCCTGATCCCGAACTCA-3’	5’-**TACGGTAGCAGAGACTTG** **GTCT**CTCCAAATTTTGTA TAGAAAGCGTCACGTACT-3’	468
	*ftsZ*	5’-**ACACTGACGACATGGTTCTACA** GGGTGGYGGWACYGGAACAGGTGC-3’	5’-**TACGGTAGCAGAGACTTG** **GTCT**TGCAGCRCTRATWGM CCTATCTTCTCC-3’	445
	*fbpA*	5’-**ACACTGACGACATGGTTC** **TACA**YGGKGCTKCAACTTAT KCTGG-3’	5’-**TACGGTAGCAGAGACTTG** **GTCT**YACTATTCTYTTYCYT GCAAARCAAGA-3’	423
PCR complementary to NGMLST	*coxA* *w*Sc2	5’-ATTGTAGCTTTGCCAGTGCT-3’	5’-TGCATAAACCATACCC ATATAACCAAA-3’	240
	*hcpA* *w*Sc2	5’-GCTGCACGCAAGGAAAATTT-3’	5’-TGCCGAAATCTTTCA TTCACA − 3’	388
*Wolbachia*-general qPCR	*fbpA*	5′-CCTGGTTCTGCTAAGTGCTTYGATATG-3′	5′- GCTGTTTCACCTTCTTTGGAAATTC-3′	89
*w*Sc1-specific qPCR	*fbpA*	5’-TGGAAGAAGCTCGCGAAATT-3’	5’-CTTCACCGCGTGGAT AAGAC-3’	81
*w*Sc2-specific qPCR	*fbpA*	5’-CTGAGGCTAAGTCTTGCGGA-3’	5’-AAAGCCGCTATATGTG CAGC-3’	121
*w*Sc1-specific qPCR	*gatB*	5’-GCACATTTGGCACTCGCT-3’	5’-CCAAAGTGACGTCAAACAGT AAGG-3’	150
*w*Sc2-specific qPCR	*gatB*	5’-GGAAAGTGGAGGGGAGATAAGT-3’	5’-TCAACAGGCAATAAATCAG GTTCT-3’	132
*w*Sc1-specific qPCR	*coxA*	5’-TCGCTCACTAAGATGCCACTA-3’	5’-CACCAGCAAGTACCGGTA AG-3’	85
*w*Sc2-specific qPCR	*coxA*	5’-TGTAGCTTTGCCAGTGCTTG-3’	5’-TACAGGATCACCACCACCAG-3’	100
*w*Sc1-specific qPCR	*hcpA*	5’-AATATGAAGGCTGCGGACCT-3’	5’-TCCCAAATTACCGCCTTTGC-3’	119
*w*Sc2-specific qPCR	*hcpA*	5’-GTGGTGTTGCAGGAGAGAATTA-3’	5’-ACGCACTTCAGAAGCAGT-3’	119
*w*Sc1-specific qPCR	*ftsZ*	5’-CAAAAGCAGCCAGAGAAGCA-3’	5’-GCACACCTTCAAAACCGAAC-3’	108
*w*Sc2-specific qPCR	*ftsZ*	5’-TCTGCACATTGGTATCAGAGGA-3’	5’-TTGCCTTGCCCATTTCACTC-3’	107

The following bioinformatic analyses were performed on the sequencing results: (i) all primers were removed; (ii) each gene was analyzed—from filtering and trimming to producing a final product amplicon sequence variant table—using the divisive amplicon denoising algorithm (DADA2); (iii) SNPs were considered true if the relative abundance of the relevant sequence was higher than 0.1% within the sample and it occurred in more than 1% of the reads of the specific gene. The bioinformatic analyses were performed in R^44^ using the *dada2* package^45^.

The *coxA* and *hcpA* primers detected less variance than other genes (see Results). To confirm that this was due to a technical limitation and not a biological phenomenon, we redesigned PCR primers based on additional *Wolbachia* sequences that were derived from the GenBank database (Table 1). The products were amplified with PCR and sequenced with the Sanger method (Macrogen, Europe). The PCR conditions are provided in the Supplementary Text S3.

### Quantitative real-time PCRs (qPCRs)

The *Wolbachia*-general qPCR quantified the total abundance of *Wolbachia* using the primers and conditions described in Flatau et al. (Table 1;^21^).

We designed variant-specific (designated as *w*Sc1 and *w*Sc2) qPCRs targeting each MLST gene (Table 1), based on sequences obtained with NGMLST (for *gatB*, *fbpA*, and *ftsZ*) and with PCR (for *coxA* and *hcpA*). Standard curves were established from samples with known numbers of *w*Sc1 and *w*Sc2 pUC-GW-Kan plasmids that were synthesized with the concatenated sequences of all five genes from each strain at GENEWIZ from Azenta Life Sciences. They were used to determine the absolute amount of *Wolbachia* strains within a pooled flea DNA extraction sample.

We confirmed the specificity of the qPCR assays by running all assays on plasmids with only one *Wolbachia* variant, the other *Wolbachia* variant, and the pooled flea DNA extraction samples containing both. Even in the highest concentrations, the cross-reaction rate never passed 0.1% for any of the genes except *hcpA w*Sc2, which showed a cross-reaction of 0.6% (Supplementary Table S1).

All specific qPCR assay conditions are provided in the Supplementary Text S3. We found out that all the variant-specific primer pairs distinguish similarly between the two strains. Thus, for the analysis of the samples from the *Wolbachia* infection manipulation, we used only qPCRs for the detection of *w*Sc1 and *w*Sc2 variants targeting *fbpA.* Subsequently, to confirm that these two assays covered all *Wolbachia* bacteria present in tested samples, we compared the cumulative results from both qPCRs on 74 flea samples with the results obtained from the *Wolbachia*-general qPCR.

### Whole genome sequencing of *Wolbachia w*Sc1 and *Wolbachia w*Sc2

We created two pools of flea extracts by assembling all the rare samples with a unique combination of variants across genes, but only one variant per gene. To do this, we gathered hundreds of flea DNA samples in our laboratory collection and subjected them to variant-specific qPCRs for each of the five MLST genes. These resulted in a total of 10 qPCRs per flea extract. After mixing the extracts that had identical MLST profiles into two unique DNA pools, we confirmed their genetic profile by subjecting each pool to another run of variant-specific qPCRs.

**Fig. 1:**
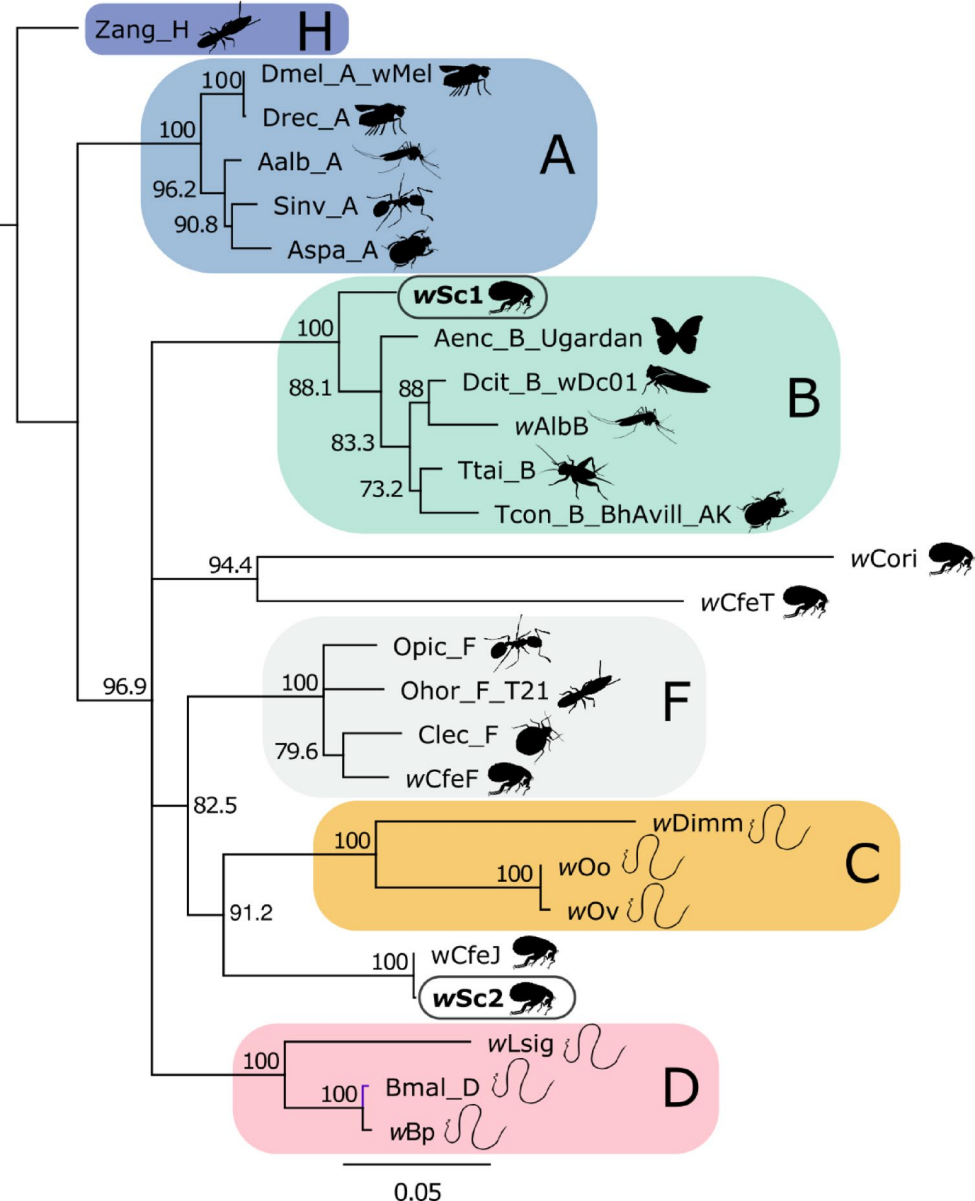
A concatenated phylogenetic tree, based on the five multilocus sequence typing (MLST) genes (*gatB*,* coxA*,* hcpA*,* fbpA*,* and ftsZ*) combined into a 1988 bp contig. The tree includes contigs of *Wolbachia* strains from different hosts (indicated by drawings) and supergroups (capital letters; more details in the Supplementary Table S2). The *Wolbachia* strains of *Synosternus cleopatrae* fleas described in this study, are marked in bold.

Between 50 and 90 ng of gDNA purified from each pool was input into the 2 S Turbo DNA Library Kit (Swift Biosciences). All reactions were carried out at 20% of the manufacturer’s recommended volumes, with dual 6-bp indexes incorporated during the final 12-cycle PCR step. The resulting libraries were pooled and sequenced on an Illumina HiSeq X Ten instrument by Psomagen (Rockville, MD) to generate 151-base paired-end reads. We removed adaptors from the Illumina reads using *trimmomatic* (v0.39) in paired-end mode with the following settings: four allowed mismatches to the seed, a palindrome clip threshold of 30, a simple clip threshold of ten, and discarding trimmed reads ≤ 30 bases. Paired Illumina reads that survived trimming were mapped to the genomes of *Wolbachia* bacteria from various arthropods (*Wolbachia w*CfeJ from *Ctenocephalides felis*, *Wolbachia w*Dci from *Diaphorina citri*, and *Wolbachia w*Con from *Tribolium confusum*) using *bowtie2* (v 2.5.4). These reference genomes were chosen from the pool of *Wolbachia* whole genomes due to the similarity of their MLST genes to the two strains we found in the *S. cleopatrae* fleas (Fig. 1). The list of all whole genomes used for comparisons and their NCBI accession numbers are provided in the Supplementary Table S2.

### Phylogenetic trees

Due to the low coverage of the whole genome sequencing data (see Results), we generated maximum-likelihood phylogenetic trees from the NGSMLST sequences. We generated a tree for each MLST target, as well as for concatenated MLST sequences. To create the concatenated tree, we prepared two contigs by concatenating sequences of the five MLST genes from each of the two sets of unique single-strain samples. Similarly, we prepared contigs for all reference *Wolbachia* bacteria included in the trees.

To cluster the two variants into the established *Wolbachia* supergroups, we added to the trees reference sequences from six out of the 19 existing supergroups^46^. These sequences, which represent the most relevant supergroups for arthropod symbionts, namely, supergroups A, B, D, F, and H, were downloaded from the *Wolbachia* MLST database (PubMLST). We aimed for a maximum of five references per supergroup. When more sequences were available, we maximized the diversity by choosing *Wolbachia* strains found in different arthropod groups. In addition, to increase the number of reference sequences from supergroups C and D, in which the *Wolbachia* documented from the *C. felis* cat flea was originally clustered^22^, we added the sequences of strains that were documented in nematodes, as well as extracted the *Wolbachia* MLST sequences from the whole genomes of *Wolbachia* strains *w*Bp, *w*Lsig, *w*Oo, *w*Ov, and *w*Dimm, which were not available in the PubMLST database. Finally, to include all the other sequences of *Wolbachia* strains that are associated with fleas, we added the MLST sequences of the other *Wolbachia* strains documented in *C. felis* (*w*CfeT, *w*CfeJ, and *w*CfeF) and those reported from *C. orientis* (*w*Cori) fleas. More details on the isolates used in the tree can be found in Supplementary Table S2.

All trees were created with the Kimura parameters model using the PhyML plugin in Geneious Prime software^47^. Transition and transversion ratio as well as gamma distribution parameters were estimated, and the proportion of invariable sites was fixed. The number of substitution rate categories was four, and topology, length, and rate were optimized. We aligned the MLST sequences by the Geneious alignment algorithm and constructed maximum likelihood phylogenetic trees with 1000 bootstrap replications.

### Statistical analyses

All the statistical analyses were performed using general linear models. To address whether only two *Wolbachia* variants coinfect the fleas (H1), we correlated the expected overall *Wolbachia* load (based on *Wolbachia*-general qPCR) and the sum of loads of the two *Wolbachia* strains (based on the variant-specific qPCRs).

To address H2 and H3, we performed analyses in which the independent variables were one of two strain-related variables, the maternal physiological age, and the interaction between the strain-related variables and age. Selecting strain-related variables was intended to tackle two distinct inquiries. The associations of the dependent variables with the strain-specific loads were used to test whether the effects are influenced by the load of each strain. The associations of the dependent variables with *Wolbachia* strain composition would reveal whether the effects are contingent upon a particular composition of the two. For the latter analysis, the strains were assigned a ‘high’ or ‘low’ load relative to the median load of each strain^48,49^, and the *Wolbachia*-free group was added.

The dependent variable of the analysis addressing H2 was the integrated index of reproductive success (RS). The index was calculated as follows: $\:RS={\sum\:}_{i}^{NF}({BS}_{F}\times\:{PS}_{F})+{\sum\:}_{l}^{NM}({BS}_{M}\times\:{PS}_{M})$, where *NF* and *NM* are the total numbers of female and male offspring, respectively, *BS*_*F*_ and *BS*_*M*_ are the body sizes of female and male offspring, respectively, and *PS*_*F*_ and *PS*_*M*_ are the survival probabilities of female and male offspring under starvation, respectively, estimated from the Kaplan-Meier survival analysis. The dependent variables in the analyses addressing H3 were the number of females, males, or the total number of offspring. We excluded fleas that did not have any offspring from these analyses. Since any of the interactions between the strain-related variables and age were not significant (Table 2), we repeated the analyses without the age effects. For post-hoc pairwise comparisons, we used the Fisher’s Least Significant Difference test.

**Table 2:** Results of the general linear models testing the effects of the different *Wolbachia* strain loads and composition (independent variables) on the integrated index of reproductive success, and on the male, female, and overall offspring number (dependent variables). Correlations with the overall offspring numbers were used to distinguish between the potential male killing and feminization mechanisms and hence were conducted only when there was an indication of reproductive manipulation. We obtained the strain composition by assigning a ‘high’ or ‘low’ load relative to the median load of each strain (a nominal variable). NA = not applicable.

Independent variables	Reproduction success	Number of male offspring per female	Number of female offspring per female	Overall offspring number per female
*Wolbachia w*Sc1 load × Maternal age	F_3,158_ = 0.502 *p* = 0.48	F_3,62_ = 2.492 *p =* 0.12	F_3,62_ = 3.271 *p =* 0.075	NA
*Wolbachia w*Sc1 load	F_1,160_ = 0.097 *p =* 0.755	F_1,64_ = 0.89 *p =* 0.349	F_1,64_ = 1.961 *p =* 0.166	NA
*Wolbachia w*Sc2 load × Maternal age	F_3,158_ = 2.285 *p =* 0.133	F_3,62_ = 0.861 *p =* 0.357	F_3,62_ = 0.102 *p =* 0.751	NA
*Wolbachia w*Sc2 load	F_1,160_ = 0.201 *p =* 0.654	F_1,64_ = 6.508 *p =* **0.013**	F_1,64_ = 0.0 *p =* 0.984	F_1,64_ = 3.998 *p =* **0.05**
Strain composition × Maternal age	F_9,152_ = 0.843 *p =* 0.5	F_7,58_ = 1.080 *p =* 0.365	F_7,58_ = 0.740 *p =* 0.533	NA
Strain composition	F_4,157_ = 2.449 *p =* **0.048**	F_3,62_ = 0.879 *p =* 0.457	F_3,62_ = 0.359 *p =* 0.783	NA

Significant results (*p* ≤ 0.05) are marked in bold.

Analyses were conducted in R/RStudio^44,50^, using the packages *stats*^44^, *agricolae*^51^, and *rstatix*^52^.

## Results

H1: Two *Wolbachia* strains coinfect individual *S. cleopatrae* fleas.

The NGMLST resulted in a total of 2,069,639 reads, distributed similarly among genes (*coxA* 3399.5 ± 3388.8, *ftsZ* 3607.3 ± 3482.5, *hcpA* 2316.7 ± 2082.6, *gatB* 3605.5 ± 3065.3, *fbpA* 1614.2 ± 1290.8). In 111 of the 128 tested female fleas, we found two variants for the *fbpA*, *ftsZ*, *gatB* genes and one variant for the *coxA* and *hcpA* genes. However, repeating the assays with more generic redesigned primers for the two latter genes resulted in the same variants as for the *fbpA*, *ftsZ*, *gatB* genes.

In the 17 (six from the laboratory and 11 from the field) remaining flea samples, only one variant was found in all the target genes. These 17 samples had identical sequences within genes. According to all phylogenetic trees, this sequence combination was clustered with *Wolbachia* supergroup B (Fig. 1 and Supplementary Figures S2–S6).

The WGS analysis of the pool of these DNA extracts resulted in a total of 13,632,934 reads and had only an overall 91.5%, identity to *Wolbachia w*CfeJ. In contrast, it was better matched to the reference *Wolbachia* sequences clustaring with supergroup B, including *Wolbachia* strains *w*Dci (overall identity 94.46%), *w*Lst (overall identity 94.55%), and *w*Con (overall identity 94.9%). We designated this strain as *w*Sc1.

To characterize the second potential coinfecting strain, designated as *w*Sc2, we conducted variant-specific qPCRs on an additional 330 males and female *Wolbachia*-positive *S. cleopatrae* fleas from our laboratory colony (see section “qPCRs”). As a result, we detected 47 flea extracts that were only amplified with the *w*Sc2-specific primers. According to all phylogenetic trees, this sequence combination is clustered with *Wolbachia w*CfeJ (Fig. 1 and Supplementary Figures S2–S6). While the association of this *Wolbachia* strain with a specific supergroup is not clear, as the different phylogenetic trees reflect slightly different associations with supergroups C, D, and F (Supplementary Figure S2–S6), based on the concatenated tree, both *w*CfeJ and *w*Sc2 are associated, to some degree, with supergroups C, D, and F, subtending supergroup C (Fig. 1 and Supplementary Figures S2–S6). The WGS analysis of the pool of fleas infected with *w*Sc2 resulted in a total of 6,224,736 reads and had an overall 99.59% identity to *w*CfeJ. In contrast, it had a lower match to the other reference *Wolbachia* sequences, namely *w*Dci (overall identity 91.15%), and *w*Tco (overall identity 91.38%). Importantly, the WGS of *w*Sc2 had only a 91.5% identity to the WGS of *w*Sc1.

Unfortunately, because of inadequate *Wolbachia* coverage in the whole genome sequencing data, which averaged less than 1% of reads, it was not feasible to assemble the *Wolbachia* genomes into FASTA files and fully complete the *w*Sc1 and *w*Sc2 genomes. Consequently, it was not possible to understand specific gene sequences and functions and utilize them for a more accurate phylogenetic tree.

**Fig. 2:**
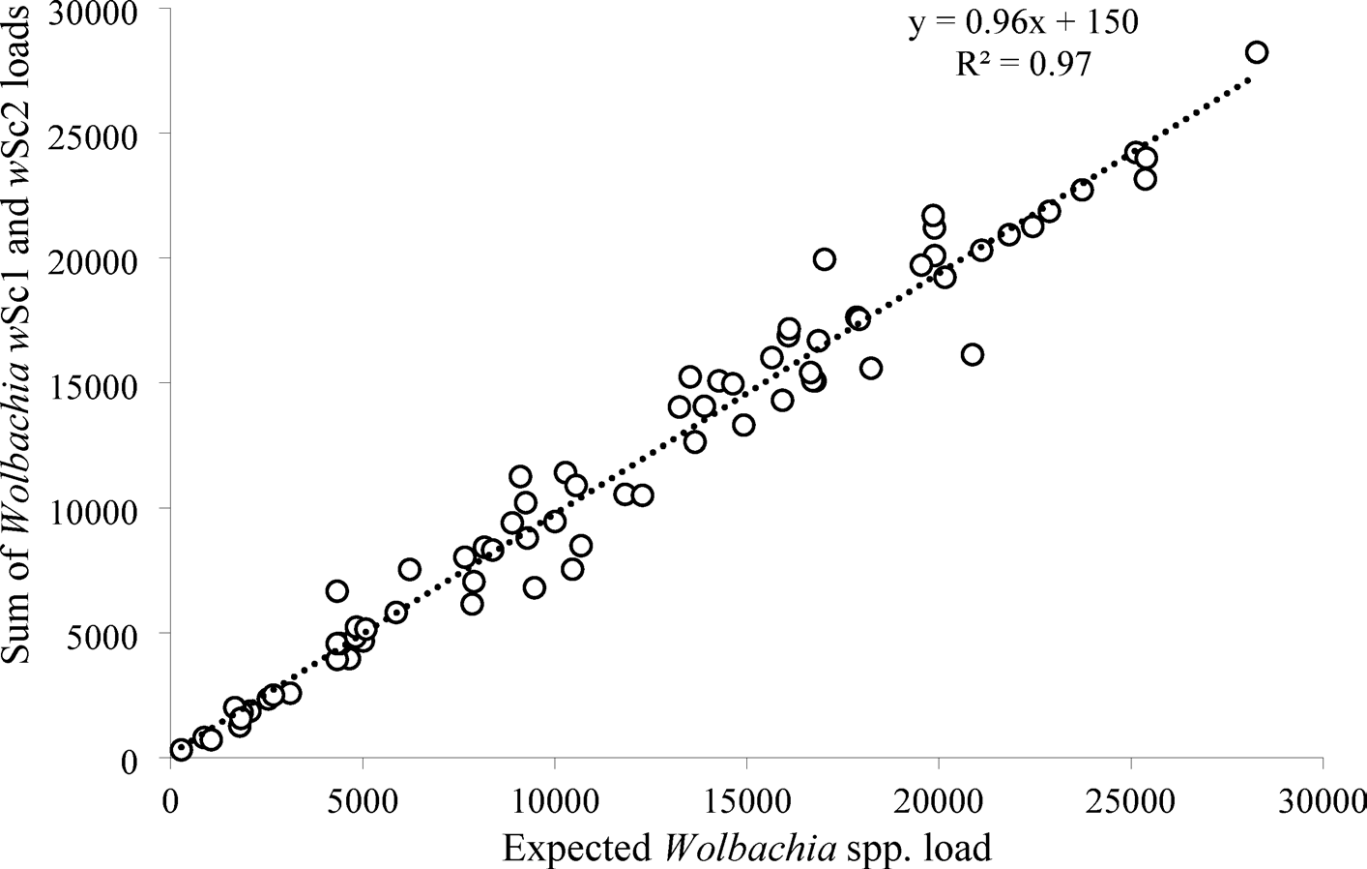
Linear correlation between the sum of bacterial load quantified by *Wolbachia* strain-specific quantitative real-time PCRs (qPCRs) and the bacterial load, as quantified by the *Wolbachia*-general qPCR assay in 74 extracts of *Synosternus cleopatrae* fleas. Axes show the number of cells per 5 µl. *w*Sc1, *Wolbachia* strain *w*Sc1; *w*Sc2, *Wolbachia* strain *w*Sc2.

DNA extracts from a total of 74 *Wolbachia-*positive female fleas from a previous study that manipulated *Wolbachia* infection^6^ were analyzed, both with the *Wolbachia-*general qPCR and the strain-specific (*w*Sc1 and *w*Sc2) primers. The overall *Wolbachia* load, as estimated by the sum of the loads calculated by the two variant-specific assays, explained 97% of the variation in the *Wolbachia* load, as estimated by the general assay (Fig. 2). Moreover, the relationship between the two methods of load estimation was highly significant (F_1,72_ = 2420, *p* = 2.2 × 10^−16^), and the slope was not significantly different from 1 (0.96, 95% CI [0.97, 1.05]), supporting the hypothesis that only *Wolbachia w*Sc1 and *w*Sc2 circulate in the *S. cleopatrae* flea populations.

H2: Evidence for a fitness advantage provided by *Wolbachia w*Sc1 but not *Wolbachia w*Sc2.

**Fig. 3:**
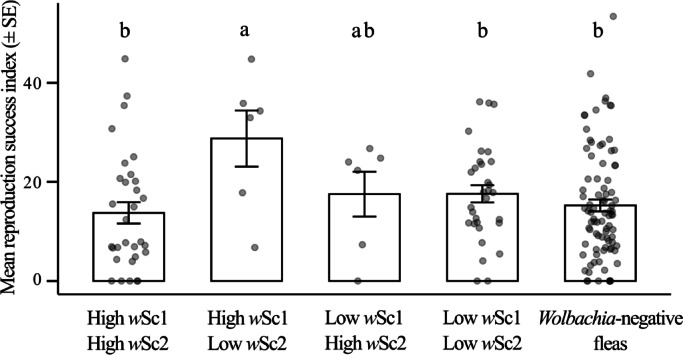
Means ± standard errors (SEs) of the integrated index of reproductive success of female fleas as a function of their *Wolbachia* strain composition (*w*Sc1 and *w*Sc2). We obtained the strain composition by assigning ‘high’ or ‘low’ status in reference to the median load of each strain. As a baseline comparison, we included in the figure and related analyses the values of the *Wolbachia*-free female fleas. The letters indicate the Fisher’s Least Significant Difference test results. *w*Sc1, *Wolbachia* strain *w*Sc1; *w*Sc2, *Wolbachia* strain *w*Sc2.

As there was no significant interaction between maternal age with any of the strain-related variables, we repeated the analyses of the integrative index of reproductive success for the entire pool of mother fleas (Table 2). There was no effect of any of the strain loads on the reproductive success index (Table 2). However, after we divided the fleas into five groups, specified based on their naturally low/high loads of *w*Sc1 and *w*Sc2 composition, we found that the reproductive success index was significantly greater in fleas that had high *w*Sc1−low *w*Sc*2* than in fleas that had high loads of both strains, low loads of both strains, or had no *Wolbachia* (Table 2; Fig. 3).

H3: Evidence for male killing induced by *Wolbachia* *w*Sc2 but not by *Wolbachia w*Sc1.

**Fig. 4:**
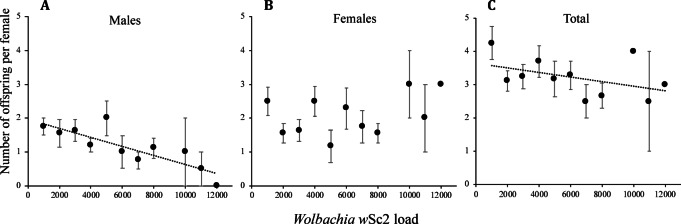
Associations between *Wolbachia w*Sc2 load (number of cells per 5 µl) and the means ± standard errors (SEs) of the numbers of male **(A)**, female **(B)**, and total offspring **(C)**. For visualization purposes, we binned bacterial loads into categories of 1000 cells. *w*Sc2, *Wolbachia* strain 2.

As there was no significant interaction between maternal age with any of the strain-related variables, we repeated the analyses of the number of females, males, and the total number of offspring for the entire pool of mother fleas (Table 2). The number of male offspring was negatively correlated with *w*Sc2 loads (Table 2; Fig. 4A), whereas the number of female offspring was not significantly correlated with any of the strain loads (Table 2; Fig. 4B). *Wolbachia w*Sc2 loads had a similar negative effect on the overall number of offspring (Table 2; Fig. 4C), supporting the mechanism of male killing rather than feminization. Both the strain ratio and the dichotomic *w*Sc1− *w*Sc2 composition were not significantly correlated with the number of male, female, or total offspring (Table 2).

## Discussion

In nature, endosymbiont coinfections are widespread. Yet, they are rarely studied in the context of the endosymbionts that exhibit continuously high incidence with unclear drivers explaining this high prevalence^53,54^. Our results provide initial evidence that contrasting effects of *Wolbachia* strains may account for their continuously high incidence in *S. cleopatrae* flea populations, and similar dynamics may occur in other endosymbiont-arthropod systems. We demonstrated that two *Wolbachia* strains circulate in natural populations of *S. cleopatrae* fleas and frequently coinfect them (see also, Supplementary Materials, Fig. S7). We further showed correlative evidence that *w*Sc1 may provide a direct fitness advantage while *w*Sc2 may cause male killing, and hence be costly for fleas. Below we discuss the results supporting the three non-mutually exclusive hypotheses in a broader context, highlight their main insights, and propose future directions to explain the continuously high endosymbiont incidence with unclear drivers in arthropods.

H1: Two *Wolbachia* strains coinfect *S. cleopatrae* flea populations.

We characterized two strains of *Wolbachia* in *S. cleopatrae* fleas. Given our successful elimination of both *Wolbachia* strains from fleas^6^, it is evident that the observed *Wolbachia* sequences represent genuine strains rather than integrated segments within the host genome. The first strain, designated as *w*Sc1, is clustered with *Wolbachia* supergroup B. This supergroup consists of *Wolbachia* bacteria found in a wide range of insects^55–57^. Considering that *Wolbachia* bacteria can cross host species barriers^55^, we speculate that at some point during their evolutionary history, *w*Sc1 was acquired by fleas from other insect groups via horizontal transmission.

The second strain, designated as *w*Sc2, is nearly identical to the *w*CfeJ strain found in *C. felis*. Based on the MLST analysis, both strains (*w*Sc2 and *w*CfeJ) are related to supergroups C, D, and F, subtending supergroup C (see also^22^). The clustering of flea-derived *Wolbachia* with filarial nematode supergroups^58,59^ may suggest that these fleas could act as vectors for *Wolbachia*-infected nematodes (e.g^60^). However, a prior study of helminth eggs in feces from 78 *Gerbillus* rodents in the same area found only two non-filarial nematode species—*Mastophorus muris* and *Syphacia* sp.—at low prevalence. To further test whether this flea species carries nematodes, we mapped its DNA reads against filarial nematode mitochondrial genomes and found no evidence for the nematode DNA (Supplementary Text S4).

Alternatively, it is possible that in the past, *w*Sc2 was horizontally transmitted from filarial nematodes to fleas, as previously suggested in other systems^60,61^. To further test the speculations about *w*Sc1 and *w*Sc2’s origin in fleas, additional phylogenomic analyses should be performed, including sequences of closely related bacteria in other host taxa, especially fleas.

H2: Evidence for a fitness advantage provided by *Wolbachia* *w*Sc1 but not *Wolbachia* *w*Sc2.

Previously, we did not find significant differences between the current reproductive success of *Wolbachia*-positive and *Wolbachia*-free (cured by antibiotic) female fleas^6^. However, the distinction between the two *Wolbachia* strains uncovered here revealed that fleas with naturally high *w*Sc1 loads and low *w*Sc2 loads had significantly higher reproductive success than those with high levels of both strains, low levels of both, or no *Wolbachia* infection. This result suggests that the potential direct advantage posed by *w*Sc1 may be masked by the possibly negative effects of *w*Sc2 on their flea hosts.

According to our phylogenetic analysis, *w*Sc1 clusters with *Wolbachia* supergroup B, which predominantly consists of reproductive manipulators^13^. Nevertheless, mutualism and reproductive parasitism by the same strain are not mutually exclusive. Gavotte et al.^62^ have shown that *Wolbachia* in *Aedes albopictus* mosquitoes shift from parasitism to mutualism in response to changes in larval density. Thus, it is possible that *w*Sc1 originally manipulated reproduction in fleas, but since it provided some indirect benefits to female fleas, their relationship has gradually become mutualistic. The nature of relationships between *w*Sc1 and their *S. cleopatrae* fleas should be further confirmed by (i) comparing the fitness of fleas that are infected solely by *w*Sc1 with those that are *Wolbachia*-free, and (ii) testing whether the *Wolbachia* cells previously detected in the flea Malpighian tubules^6^ are of the *w*Sc1 strain. The latter could indicate that *w*Sc1 plays a nutritional role in fleas.

H3: Evidence for male killing induced by *Wolbachia* *w*Sc2 but not *Wolbachia* *w*Sc1.

Female fleas harboring high loads of *w*Sc2 had significantly fewer male and total offspring than females with low loads of *w*Sc2. Interestingly, there was no evidence for sex ratio distortion in *Wolbachia*-positive versus *Wolbachia*-free colonies in the previous infection manipulation study by Flatau et al.^6^. This could indicate that male killing is density-dependent, which was mitigated in the previous study, as the *Wolbachia*-positive colony consists of females with varying *w*Sc2 loads. The reduction in both male and total offspring observed here suggests the occurrence of male killing rather than feminization induced by *Wolbachia*. This is because the latter mechanism is not expected to affect the overall offspring number. As in other host species, *Wolbachia* in *S. cleopatrae* is transmitted maternally^6^, suggesting that males are evolutionary dead ends for these endosymbiotic bacteria. Therefore, in theory, male killing should enhance *Wolbachia* transmission if the fitness of female fleas increases because of the death of their male siblings^14^. In *S. cleopatrae* fleas, a reduction in male offspring might reduce the intraspecific competition that is known to occur between larvae^63,64^, adults^65^, or both.

Contrary to *w*Sc2, *w*Sc1 loads were not associated with male, female, or overall offspring numbers. Coinfecting *Wolbachia* strains may affect their host in similar ways but the intensity of their effects may differ^66,67^. Alternatively, coinfecting *Wolbachia* strains can play distinct roles in their host biology. For example, it was shown experimentally that the *Asobara tabida* wasp harbors three strains of *Wolbachia*, two of which induce CI, while the third one is an obligate endosymbiont necessary to achieve oogenesis^68,69^. Likewise, genomic analyses suggest that in *C. felis* fleas, *w*CfeT is a nutritional endosymbiont, while the coinfecting *w*CfeJ induces CI^22^. The nature of the relationships between *w*Sc2 and their *S. cleopatrae* fleas should be further tested by (i) comparing the offspring number and sex ratio of fleas that are experimentally infected solely with *w*Sc2 with those that are *Wolbachia*-free and (ii) quantifying larvae and adult intraspecific competition in *w*Sc2-infected offspring that are either supplemented artificially with male fleas or not.

*Broad implications of Wolbachia coinfection in flea populations*.

This study provides initial indications that the intricate tripartite relationship between two *Wolbachia* strains and their flea host may be the key toward a better understanding of both the continuously high incidence of *Wolbachia* in female but not in male fleas, and the high strain coinfection rates observed in natural flea populations (Supplementary Materials, Fig. S7). In particular, we collected evidence suggesting that *Wolbachia w*Sc1 may be responsible for the continuously high incidence observed in female fleas, whereas male killing by *Wolbachia w*Sc2 may explain the low abundance of *Wolbachia* in the male fleas. Such sex-dependent interactions between a host and its coinfecting endosymbionts may promote the coexistence of the two strains.

The high percentage of flea eggs infected by both *Wolbachia* strains (Supplementary Fig. S8), along with the stable presence of coinfection observed in both laboratory and natural settings (Supplementary Fig. S7), suggests that these strains are vertically cotransmitted. This adds to the growing evidence of widespread coinfection among vertically transmitted symbionts^23,70–72^. While such transmission typically reduces endosymbiont diversity due to bottlenecks, theoretical models and empirical studies suggest that CI^73^, functional complementarity^74^, and interactions between beneficial symbionts and reproductive manipulators^75–77^ can stabilize coinfections over time^23^. Our findings align with this latter scenario, suggesting a stable long-term coexistence—likely facilitated by cooperation^78,79^—between the *Wolbachia* strains *w*Sc1 and *w*Sc2 in natural flea populations. Future studies should explore the nature of associations between these two strains in their flea host and whether they are context-dependent^80^.

Taken together, our findings contribute to a broader understanding of how endosymbiont diversity is preserved over time despite potential host-symbiont conflicts and changing ecological conditions and highlight the need to pay attention to multiple mechanisms and players under various conditions.

## Electronic supplementary material

Below is the link to the electronic supplementary material.

Supplementary Material 1

## Data Availability

All data supporting the findings of this study are available within the paper and its Supplementary Information. MLST raw sequencing data generated in this study have been deposited in the NCBI Sequence Read Archive (SRA) and are available under BioProject (accession number: PRJNA1281168) at https://www.ncbi.nlm.nih.gov/bioproject/1281168.
