# Supplementary material for: Continuously high Wolbachia incidence in flea populations may result from dual-strain infections with divergent effects
Source: Sci Rep. 2025 Jul 1;15:21720. doi: 10.1038/s41598-025-09403-2 (PMC12218854; doi:10.1038/s41598-025-09403-2)
Supplement: Supplementary file 1 — Supplementary Material 1 [file 41598_2025_9403_MOESM1_ESM.docx]

## Supplementary Materials

## *Supplementary Text*

# Text S1. Flea origins

To explore the *Wolbachia* genetic variation of field-collected *Synosternus cleopatrae* fleas, we used DNA extracts from fleas that were collected during a field survey in 2011 at three sites across the northwestern Negev Desert’s sands in Israel (Site 1, 34°37'E 30°58'N; Site 2, 34°38'E 30°58'N, and Site 3, 34°41'E 30°59'N; [1]). We used live traps to capture rodents. At each site, we randomly selected two female and two male *Gerbillus andersoni* and *G. pyramidum* rodents, and from each of these individuals, we randomly selected four female fleas. This design resulted in a total of 96 *S. cleopatrae* female fleas (3 sites × 2 hosts × 4 hosts per species × 4 fleas per host).

To explore the *Wolbachia* genetic variation of *S. cleopatrae* fleas that were bred under laboratory conditions, we used DNA extracts from our laboratory colonies of *S. cleopatrae* fleas that were raised on *G. andersoni* and *G. pyramidum* rodents [2]. Here too, we randomly selected two female and two male *G*. *andersoni* and *G. pyramidum* rodents, and from each of these individuals, we randomly selected four female fleas. This design resulted in a total of 32 *S. cleopatrae* female fleas (2 hosts × 4 hosts per species × 4 fleas per host).

Fleas for the *Wolbachia* manipulation experiment also originated from our laboratory colony. The establishment of the *Wolbachia*-positive and *Wolbachia*-free groups is fully described in Flatau et al. [2]. In short, larval *S. cleopatrae* fleas were randomly assigned to either a tetracycline antibiotic or served as a control group (*Wolbachia*-positive fleas). Then, to reduce the direct effect of the antibiotics on the fleas and to allow the fleas to restore their natural bacterial community, the adult fleas emerging from both treatment groups were reared separately on rodents for an additional three generations (150 days) without antibiotics.

To assess the persistence of coinfection by *Wolbachia* *w*Sc1 and *w*Sc2 strains in S. *cleopatrae* populations, DNA samples of female fleas collected on various occasions from the field and laboratory colonies were tested with strain-specific qPCRs targeting fbpA. In the field, fleas were obtained from wild rodents captured during the 2011 sampling (Site 1, 34°37'E 30°58'N; Site 2, 34°38'E 30°58'N, and Site 3, 34°41'E 30°59'N), as well as from a nearby site in 2023 (30°55'N 34°25'E). Laboratory fleas, on the other hand, were collected at various intervals between October 2016 and June 2023 from our control colony of *S. cleopatrae*, which were raised on *G. andersoni* rodents and known to be *Wolbachia*-positive.

To evaluate the percentage of each *Wolbachia* strain in flea eggs, 23 females and 29 males fleas from the same cohort were fed on *Gerbillus andersoni* rodents. After three days of feeding and mating, individual female fleas were placed in separate vials for egg laying. After 48 hours, 31 eggs were collected. Each egg was individually extracted, and the extracts were analyzed by qPCR using the same protocols applied to adult fleas.

# Text S2. Details on the previous infection manipulation study [2]

Fleas from the *Wolbachia*-positive and *Wolbachia*-free treatment groups (see Text S1) were randomly divided into six subgroups, each subjected to either five or 10 days of feeding and mating (2 h/d of feeding and mating on the host). Then, 88 *Wolbachia*-free (43 and 45 after five and 10 days of feeding and reproduction, respectively) and 79 (39 and 40 after five and 10 days of feeding and reproduction, respectively) *Wolbachia*-positive female fleas were collected into separate plastic vials, where the individual parent females were allowed to lay eggs for 24 h. From day 30 on, the vials were monitored daily for newly emerged offspring that were sexed and measured. We used the mean length of the two tibias raised to the power of three as an approximation of each offspring’s body size. We then incubated the newly emerged offspring and daily monitored them until their death. The number of days until their death was used to quantify their survival rate under starvation. At the end of the experiment, we subjected the parent fleas to DNA extraction.

# Text S3. PCR and qPCR conditions

## *Next-generation multilocus sequence typing (NGMLST) PCR*

For each gene, conventional PCR amplifications were carried out in a total reaction volume of 25 µl obtained by adding 12.5 μl 2× PCRBIO mix (HS Taq Mix Red), 0.2 μl of each primer (800 nM), 7.1 μl of water, and 5 μl of a template. The PCR conditions for all the genes were 5 min at 95°C, followed by 8 cycles of 30 sec at 95°C, and 30 sec at gene-specific temperatures (60°C for all genes except *gatB*, for which the temperature was 55°C). We ended the reactions by running them for 5 min at 72°C and holding them on completion at 8°C.

## *NGMLST complementary PCR*

For *coxA* and *hcpA* genes, conventional PCR amplifications were carried out in a total volume of 25 μl obtained by adding 12.5 μl 2× PCRBIO mix (HS Taq Mix Red), 0.2 μl of each primer (800 nM), 7.1 μl of water, and 5 μl of a template. The PCR conditions for all the genes were 5 min at 95°C, followed by 35 cycles of 30 sec at 95°C, and 30 sec at 60°C. We ended the reactions by running them for 5 min at 72°C and holding them on completion at 8°C.

## Wolbachia*-general qPCR*

qPCR amplifications were carried out in a total reaction volume of 20 μl obtained by adding 10 μl Bio-Rad SYBR® Green Master Mix, 1 μl of each primer in the concentration of 400 nM, 3 μl of water, and 5 μl of template. The qPCR conditions were 3 min at 95°C followed by 35 cycles of 5 sec at 95°C, and 20 sec at 63.3°C.

## *Strain-specific qPCR*

For each gene, qPCR amplifications were carried out in a total reaction volume of 20 μl obtained by adding 10 μl Bio-Rad SYBR® Green Master Mix, 1 μl of each primer in specific concentration for a gene and strain (Table S1), 3 μl of water, and 5 μl of template. The qPCR conditions for all genes were 3 min at 95°C followed by 35 cycles of 5 sec at 95°C, and 20 sec at gene-specific temperature (Table S1).

**Text S4. Methodology for searching nematode sequences in flea DNA**

Our finding that the *Wolbachia* *w*Sc2 strain from the *S. cleopatrae* fleas clad along with nematode supergroups may suggest that fleas might harbor *Wolbachia*-infected nematodes. To investigate the presence of nematode sequences in flea DNA, we mapped the raw reads of the fleas’ DNA extracts (see “whole genome sequencing of *w*Sc1 and *w*Sc2 section in the main document) to the complete mitochondrial genomes of three filarial nematode species that parasitize rodents, humans, and other mammals: *Acanthocheilonema viteae*, *Mansonella ozzardi*, and *Setaria digitata*. Raw data files were trimmed to eliminate adaptors and low-quality sequences using BBDuk (version 39.01, BBTools package). The quality of the trimmed reads was evaluated with FastQC. The processed reads from each sample (*w*Sc1 and *w*Sc2) were then mapped to the full mitochondrial genomes with BBMap (version 39.01, BBTools package), using a minimum identity threshold of 70%. This threshold was selected to capture sequences with moderate divergence while filtering out random noise. Despite this conservative approach, no indications of nematode DNA were found in any of the samples.

**References**

1. Messika, I., Garrido, M., Kedem, H., China, V., Gavish, Y., Dong, Q. *et al.* (2017). From endosymbionts to host communities: factors determining the reproductive success of arthropod vectors. *Oecologia*, *184*, 859-871.
2. Flatau, R., Segoli, M. and Hawlena, H. (2021). *Wolbachia* endosymbionts of fleas occur in all females but rarely in males and do not show evidence of obligatory relationships, fitness effects, or sex-distorting manipulations. *Frontiers in Microbiology*, *12*, 649248.

## *Supplementary Tables*

**Table S1.** qPCR primer conditions and cross-reactivity rate per gene and strain.

| **Target strain** | **Target gene** | **Primer concentration (nM)** | **Annealing**  **temperature (**°C**)** | **Cross-reactivity rate (%)** |
| --- | --- | --- | --- | --- |
| *w*Sc1 | *gatB* | 600 | 58 | 0.0015 |
| *w*Sc2 | *gatB* | 600 | 58 | 0.0000 |
| *w*Sc1 | *coxA* | 800 | 60 | 0.0018 |
| *w*Sc2 | *coxA* | 600 | 61.5 | 0.0001 |
| *w*Sc1 | *hcpA* | 800 | 62 | 0.0000 |
| *w*Sc2 | *hcpA* | 800 | 60 | 0.5912 |
| *w*Sc1 | *ftsZ* | 800 | 62 | 0.0903 |
| *w*Sc2 | *ftsZ* | 600 | 60 | 0.0327 |
| *w*Sc1 | *fbpA* | 800 | 61.5 | 0.0000 |
| *w*Sc2 | *fbpA* | 600 | 60 | 0.0000 |

**Table S2.** A list of *Wolbachia* strains used in the phylogenetic trees and whole genomic sequences comparisons. N/A, not assigned.

| **Strain name** | **Host name** | ***Wolbachia***  **supergroup** | **Source** | **Accession number or ID** |
| --- | --- | --- | --- | --- |
| Dmel_A_wMel | *Drosophila melanogaster* | A | PubMLST | 1 |
| Sinv_A | *Solenopsis invicta* | A | PubMLST | 2 |
| Aspa_A | *Acromis sparsa* | A | PubMLST | 3 |
| Drec_A | *Drosophila recens* | A | PubMLST | 9 |
| Tcon_B_BhAvill_AK | *Tribolium confusum* | B | PubMLST | 20 |
| Aenc_B_Ugardan | *Acraea encedon* | B | PubMLST | 22 |
| Ttai_B | *Teleogryllus taiwanemma* | B | PubMLST | 25 |
| Bmal_D | *Brugia malayi* | D | PubMLST | 37 |
| Opic_F | *Ocymyrmex picardi* | F | PubMLST | 149 |
| Zang_H | *Zootermes angusticollis* | H | PubMLST | 207 |
| Aalb_A | *Aedes albopictus* | A | PubMLST | 12 |
| Dcit_B_wDc01 | *Diaphorina citri* | B | PubMLST | 267 |
| *w*AlbB | *Aedes albopictus* | B | PubMLST | 1847 |
| *w*Cori | *Ctenocephalides orientis* | N/A | NCBI | CP116769.1 |
| *w*CfeT | *Ctenocephalides felis* | N/A | NCBI | CP051156.1 |
| *w*Lsig | *Litomosoides sigmodontis* | D | NCBI | CP046577 |
| *w*Bp | *Brugia pahangi* | D | NCBI | CP050521.1 |
| Ohor_F_T21 | *Odontotermes horni* | F | PubMLST | 253 |
| Clec_F | *Cimex lectularius* | F | PubMLST | 36 |
| *w*CfeF | *Ctenocephalides felis* | F | NCBI | CP116767.1 |
| *w*CfeJ | *Ctenocephalides felis* | N/A | NCBI | CP051157.1 |
| *w*Dimm | *Dirofilaria immitis* | C | NCBI | CP046578 |
| *w*Oo | *Onchocerca ochengi* | C | NCBI | NC_018267.1 |
| *w*Ov | *Onchocerca volvulus* | C | NCBI | HG810405.1 |
| *w*Dci | *Diaphorina citri* | B | NCBI | CP048819.1 |
| *w*Con | *Tribolium confusum* | B | NCBI | PRJNA767570 |

## *Supplementary Figures*

**Figure S1.** Study approach and rationale according to the three tested non-mutually exclusive hypotheses (H1−H3). Orange boxes with a solid outline, blue boxes with a dashed outline, and green boxes with a dotted outline represent questions, methods, and results, respectively. NGMLST, next-generation multilocus sequence typing; qPCR, real-time quantitative polymerase chain reaction; *w*Sc1, *Wolbachia* strain *w*Sc1; *w*Sc2, *Wolbachia* strain *w*Sc2; WGS, whole genome sequencing.


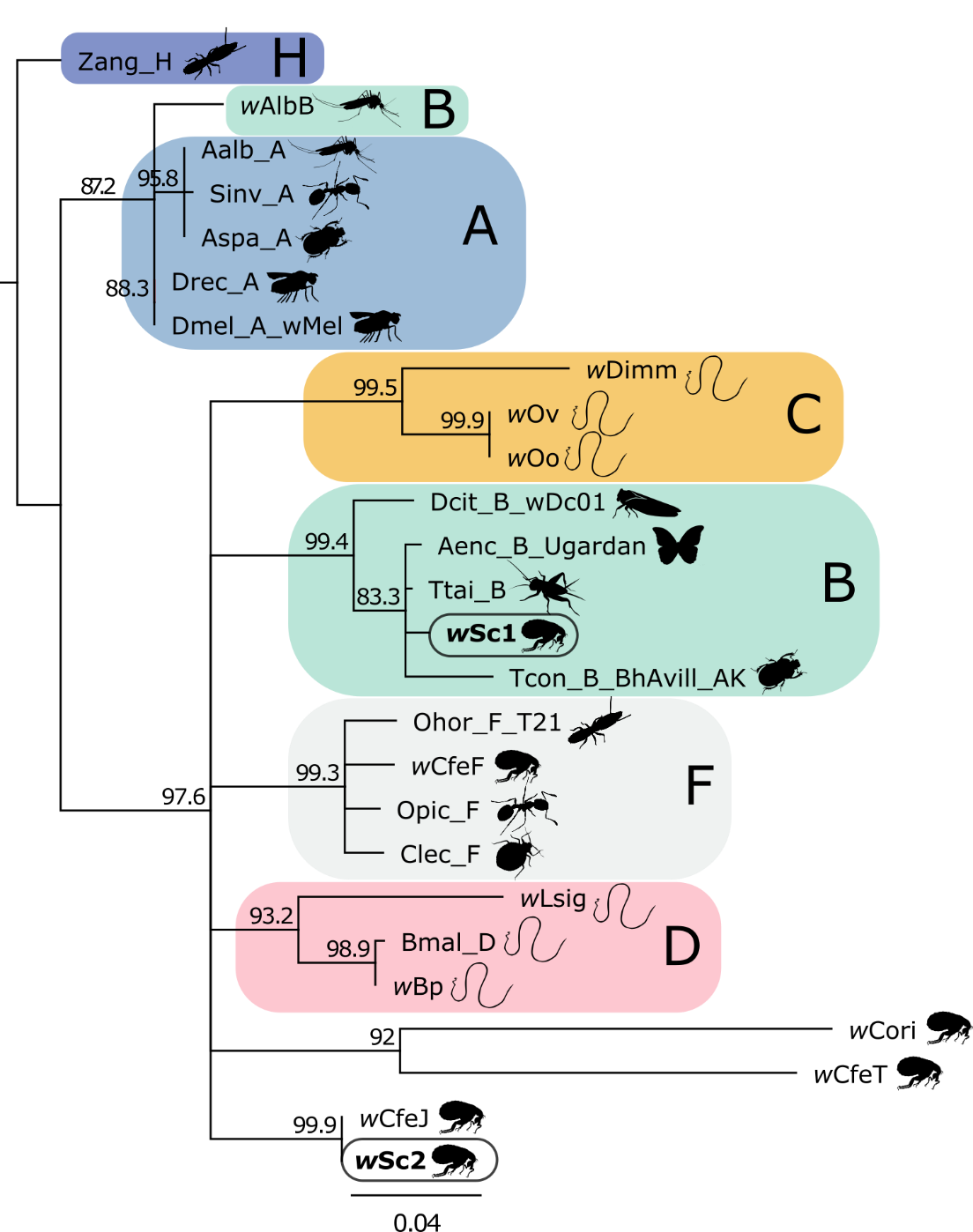


**Figure S2.** Phylogenetic *Wolbachia* tree based on the *coxA* gene representing 387 bp*.* The tree includes sequences of *Wolbachia* strains from different hosts (indicated by drawings) and supergroups (capital letters; more details in Table S2). The *Wolbachia* strains of *Synosternus cleopatrae* fleas described in this study, are marked in bold.


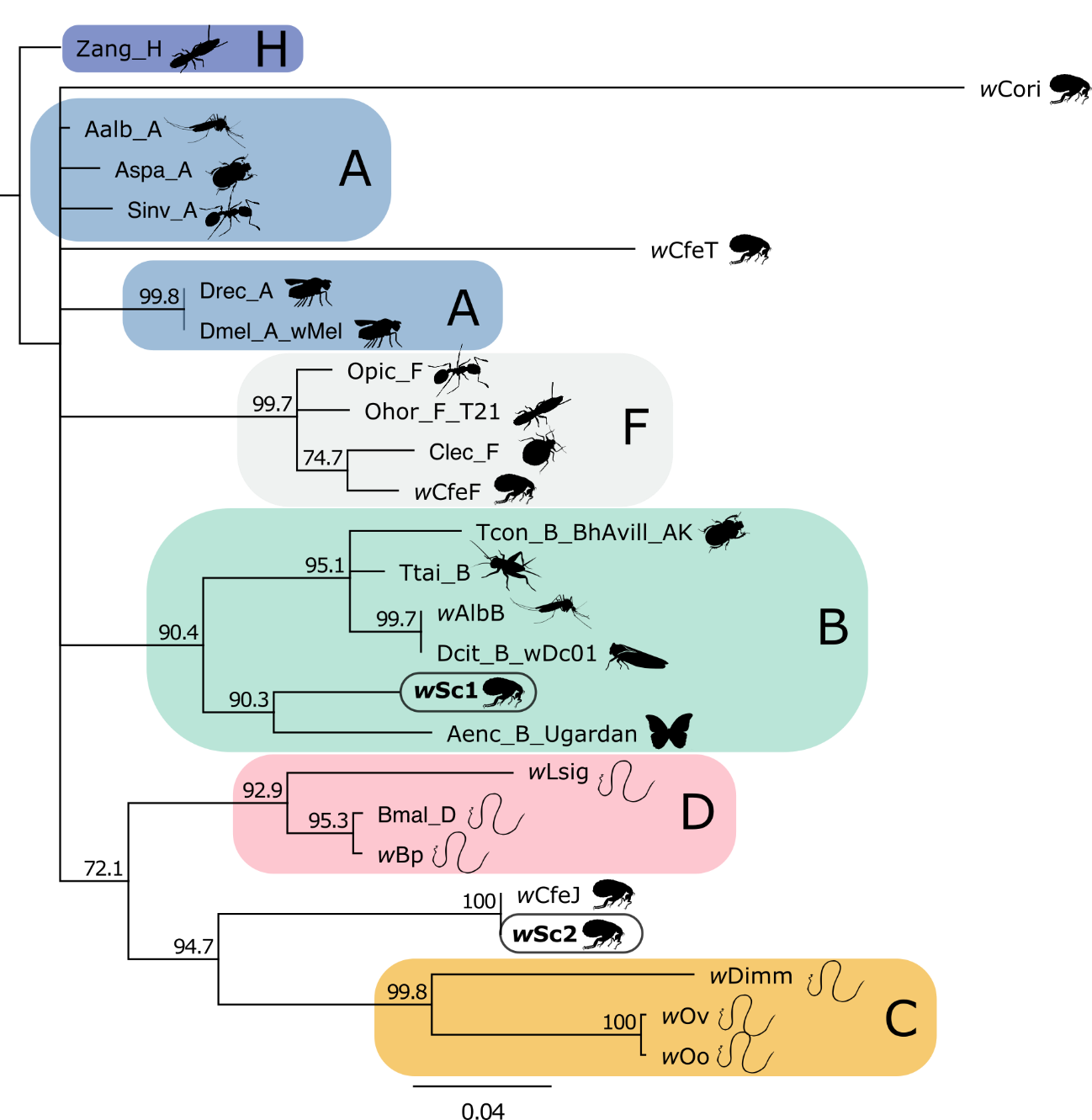


**Figure S3**. Phylogenetic *Wolbachia* tree based on the *fbpA* gene representing 419 bp*.* The tree includes sequences of *Wolbachia* strains from different hosts (indicated by drawings) and supergroups (capital letters; more details in Table S2). The *Wolbachia* strains of *Synosternus cleopatrae* fleas described in this study, are marked in bold.


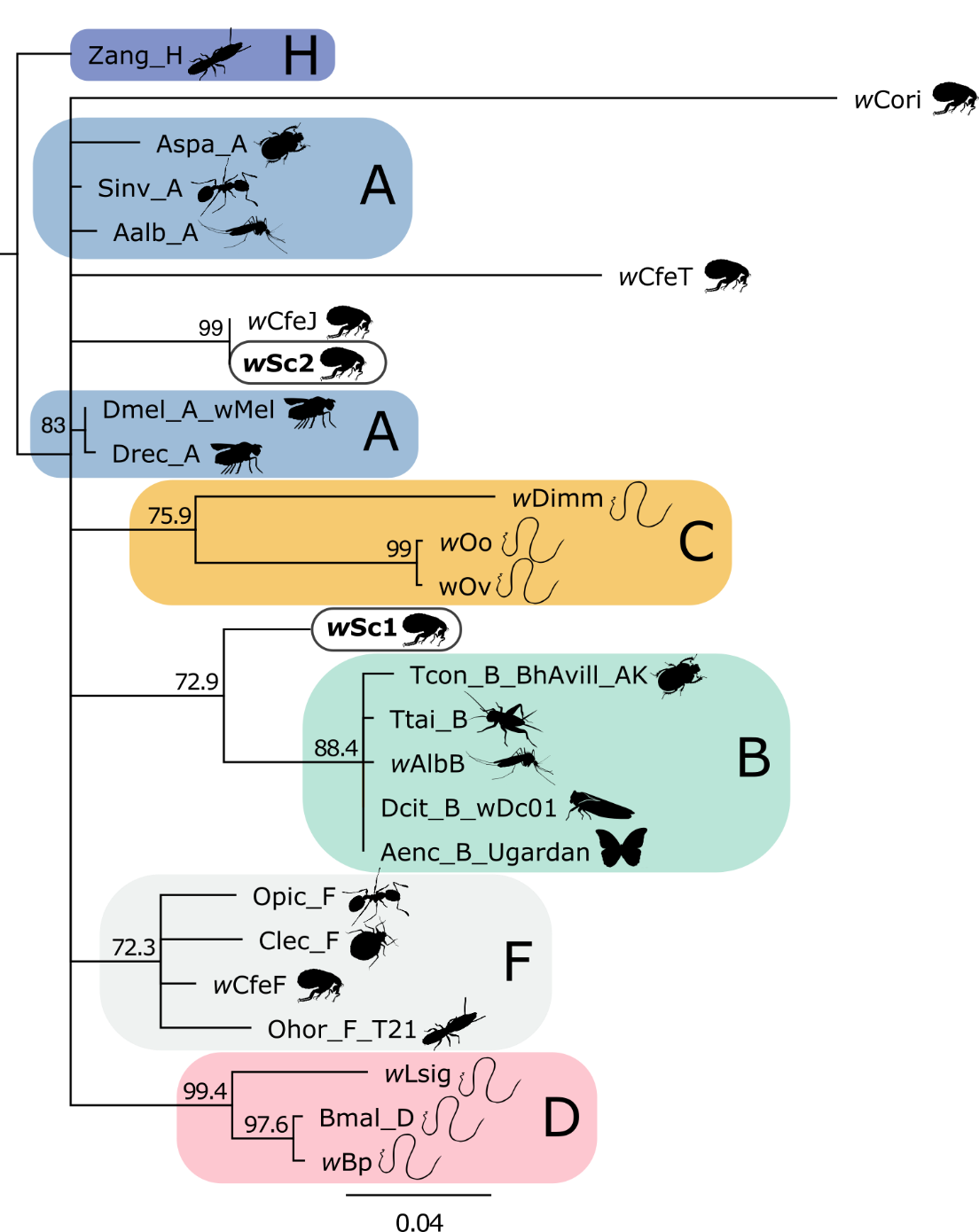


**Figure S4.** Phylogenetic *Wolbachia* tree based on the *ftsZ* gene representing 396 bp*.* The tree includes sequences of *Wolbachia* strains from different hosts (indicated by drawings) and supergroups (capital letters; more details in Table S2). The *Wolbachia* strains of *Synosternus cleopatrae* fleas described in this study, are marked in bold.


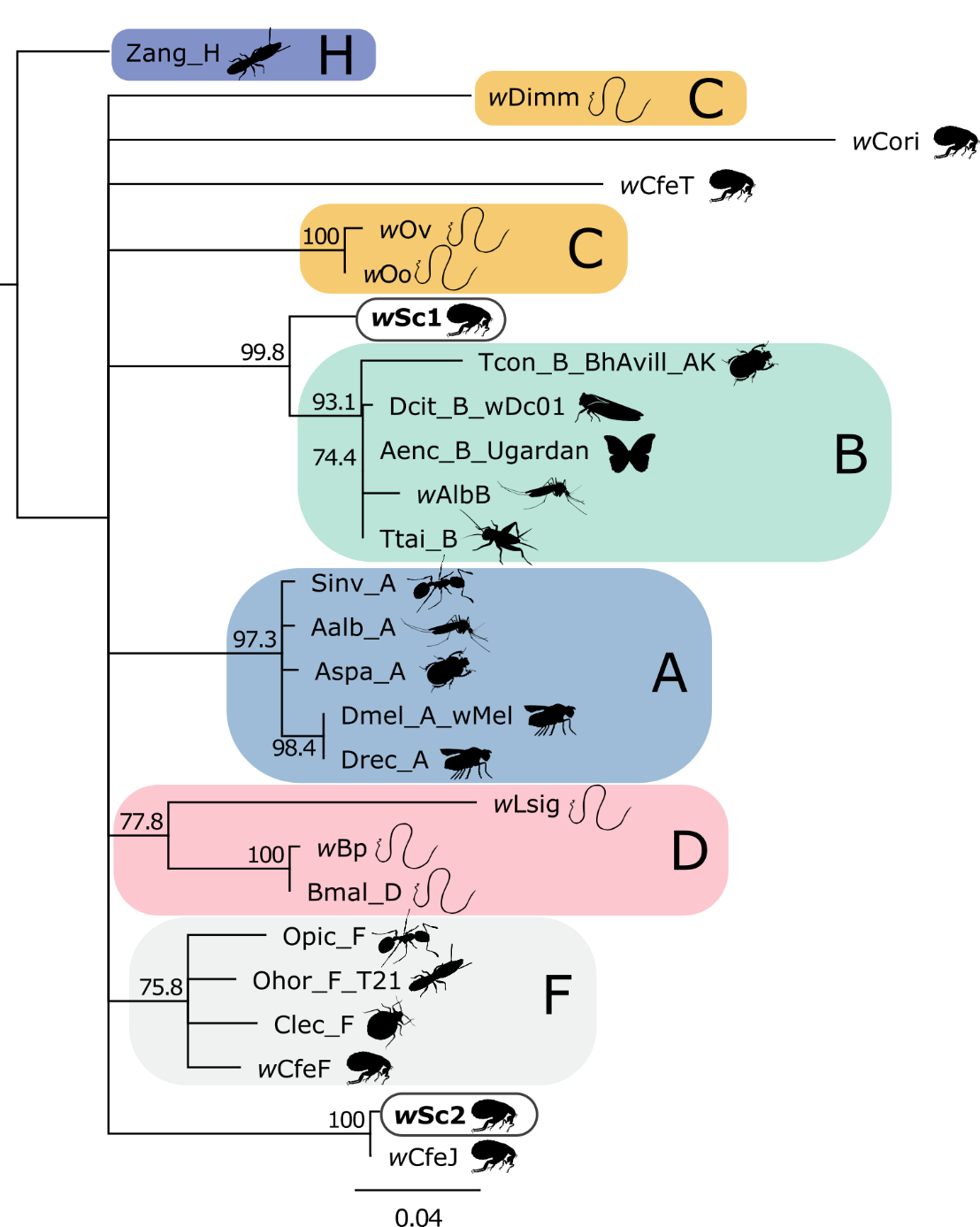


**Figure S5**. Phylogenetic *Wolbachia* tree based on the *gatB* gene representing 369 bp*.* The tree includes sequences of *Wolbachia* strains from different hosts (indicated by drawings) and supergroups (capital letters; more details in Table S2). The *Wolbachia* strains of *Synosternus cleopatrae* fleas described in this study, are marked in bold.


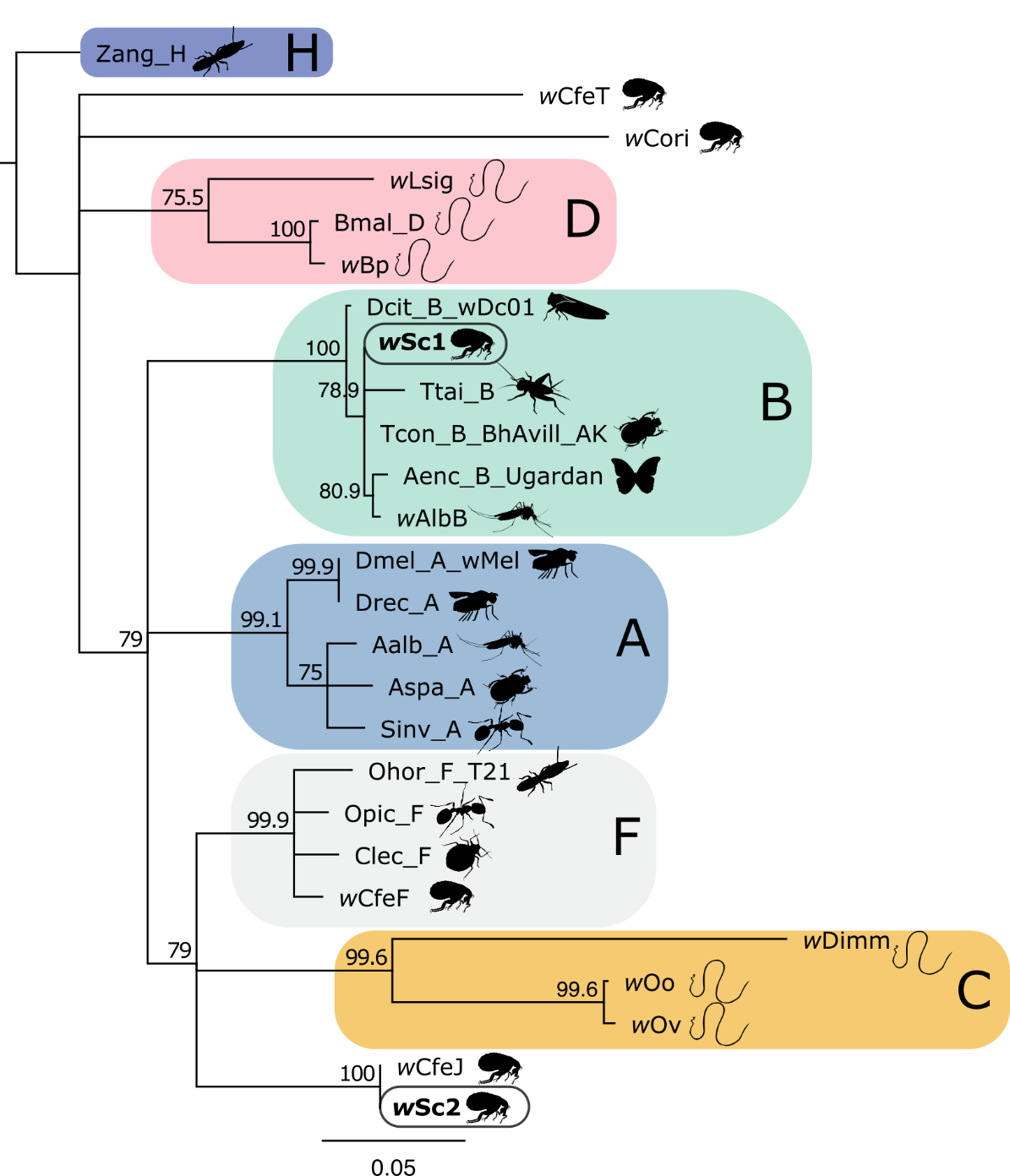


**Figure S6**. Phylogenetic tree of *Wolbachia* based on the *hcpA* gene representing 417 bp*.* The tree includes sequences of *Wolbachia* strains from different hosts (indicated by drawings) and supergroups (capital letters; more details in Table S2). The *Wolbachia* strains of *Synosternus cleopatrae* fleas described in this study, are marked in bold.

**Figure S7.** Continuously high incidence of coinfection by *Wolbachia* *w*Sc1 and *w*Sc2 strains in *Synosternus cleopatrae* female fleas over time. Mean (± SE) coinfection probability in fleas collected on various occasions from the field (filled bars) and laboratory (empty bars) colonies. The numbers above the bars indicate sample sizes. Refer to **Text S1** for additional information regarding the methodology employed in these sampling events.

Percents of *Wolbachia*-infected eggs

*Wolbachia* load

A

B


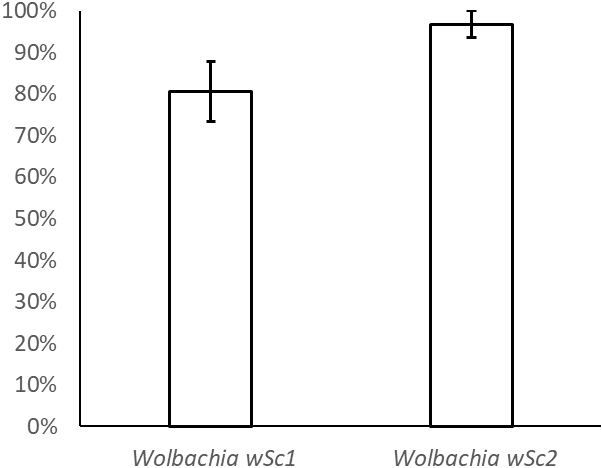

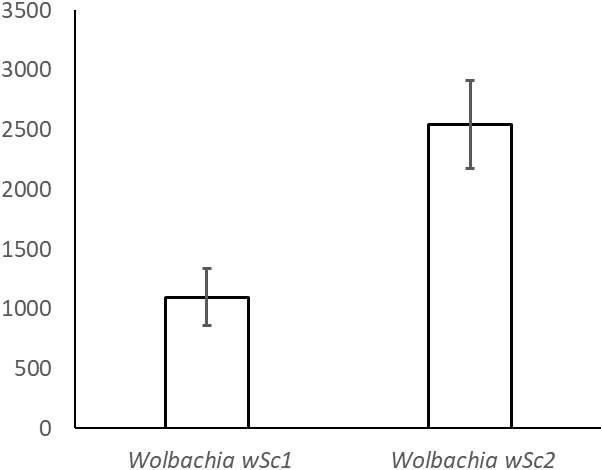


Fig. S8. Mean values and standard errors for (A) the percentages and (B) the loads (measured as the number of cells per 5 μl of DNA) of *Wolbachia* strains *w*Sc1 and *w*Sc2 in *Synosternus cleopatrae* flea eggs. The comparatively lower percentages of *Wolbachia* wSc1 may be due to its reduced bacterial load, which increases the likelihood of falling below the detection threshold of qPCR.
